# The UMBRELLA SIOP–RTSG 2016 Wilms tumour pathology and molecular biology protocol

**DOI:** 10.1038/s41585-018-0100-3

**Published:** 2018-10-11

**Authors:** Gordan M. Vujanić, Manfred Gessler, Ariadne H. A. G. Ooms, Paola Collini, Aurore Coulomb-l’Hermine, Ellen D’Hooghe, Ronald R. de Krijger, Daniela Perotti, Kathy Pritchard-Jones, Christian Vokuhl, Marry M. van den Heuvel-Eibrink, Norbert Graf, on behalf of the International Society of Paediatric Oncology–Renal Tumour Study Group (SIOP–RTSG)

**Affiliations:** 1 Department of Pathology, Sidra Medicine, Doha, Qatar; 2 Theodor-Boveri-Institute/Biocenter, Developmental Biochemistry, Wuerzburg University, Wuerzburg, Germany; 3 Comprehensive Cancer Center Mainfranken, Wuerzburg University, Wuerzburg, Germany; 4 Princess Maxima Centre for Pediatric Oncology, Utrecht, Netherlands; 5 Department of Diagnostic Pathology and Laboratory Medicine, Fondazione IRCCS Istituto Nazionale dei Tumori, Milan, Italy; 6 Sorbonne Université, Department of Pathology, Hopitaux Universitaires Est Parisien, Paris, France; 7 Department of Pathology, Oslo University Hospital, Rikshospitalet, Oslo, Norway; 8 Molecular Bases of Genetic Risk and Genetic Testing Unit, Department of Preventive and Predictive Medicine, Fondazione IRCCS Istituto Nazionale dei Tumori, Milan, Italy; 9 Great Ormond Street Institute of Child Health, University College London, London, UK; 10 Kiel Paediatric Tumour Registry, Department of Paediatric Pathology, University Hospital of Kiel, Kiel, Germany; 11 Department of Paediatric Oncology & Haematology, Saarland University, Homburg, Germany

**Keywords:** Paediatric cancer, Renal cancer, Pathology, Molecular biology

## Abstract

On the basis of the results of previous national and international trials and studies, the Renal Tumour Study Group of the International Society of Paediatric Oncology (SIOP–RTSG) has developed a new study protocol for paediatric renal tumours: the UMBRELLA SIOP–RTSG 2016 protocol (the UMBRELLA protocol). Currently, the overall outcomes of patients with Wilms tumour are excellent, but subgroups with poor prognosis and increased relapse rates still exist. The identification of these subgroups is of utmost importance to improve treatment stratification, which might lead to reduction of the direct and late effects of chemotherapy. The UMBRELLA protocol aims to validate new prognostic factors, such as blastemal tumour volume and molecular markers, to further improve outcome. To achieve this aim, large, international, high-quality databases are needed, which dictate optimization and international harmonization of specimen handling and comprehensive sampling of biological material, refine definitions and improve logistics for expert review. To promote broad implementation of the UMBRELLA protocol, the updated SIOP–RTSG pathology and molecular biology protocol for Wilms tumours has been outlined, which is a consensus from the SIOP–RTSG pathology panel.

## Abstract (web summary)

In this Consensus Statement, the pathology panel of the International Society of Paediatric Oncology–Renal Tumour Study Group (SIOP–RSTG) present the pathology and molecular biology protocol for Wilms tumours in the UMBRELLA SIOP-RTSG 2016 protocol.

## Introduction

Paediatric renal tumours account for ~6% of all paediatric malignant tumours, of which ~90% are Wilms tumours (nephroblastoma)^1^. Other renal non-Wilms tumours are rare entities and include mesoblastic nephroma, clear cell sarcoma of the kidney, rhabdoid tumour of the kidney, renal cell carcinoma and few other, even rarer tumour types^1^. The Renal Tumour Study Group of the International Society of Paediatric Oncology (SIOP–RTSG) has developed a new study for all renal tumours of childhood, the UMBRELLA SIOP–RTSG 2016 protocol (the UMBRELLA protocol)^2,3^, on the basis of the experiences from SIOP–2001 and the UK Improving Population Outcomes for Renal Tumours of Childhood (IMPORT) 2013 studies^2–4^. The aim of the UMBRELLA protocol is to collect all clinical, biological and outcomes data from children with primary renal tumours in a comprehensive data registry, with central review of diagnostics (radiology, pathology and surgery), standardized biobanking and precise treatment recommendations for the most common paediatric renal tumours. Molecular biology research within the protocol is primarily focused on the validation of the prognostic value of 1q gain, which might lead to a more personalized treatment approach (Box 1). Consequently, short-term and long-term outcomes might be improved for all children with renal tumours by increasing survival, but also by reducing treatment in specific subgroups, resulting in diminished direct and late adverse effects. Timely genetic analysis and step-wise extension to additional targets such as *TP53* (refs^5–7^) or several of the newly identified driver candidates for stratification and inclusion of liquid biopsies might help to reach this goal^8–10^.

The UMBRELLA protocol includes updated guidelines for pathologists for the handling and processing of tissue as well as criteria that are important for postoperative histological classification, staging and treatment stratification. These recommendations were established by a consensus of pathology experts within the SIOP–RTSG (chaired by G. M. Vujanić and I. Leuschner). As well as the aim of validating 1q gain as a prognostic marker^11,12^, one of the main focuses of the UMBRELLA protocol is assessment of the independent prognostic value of the absolute volume (rather than the percentage) of the blastemal component that survives preoperative chemotherapy^13–15^. Furthermore, in order to optimize pathological diagnostics and treatment, rapid central pathology review will be obligatory to enable feedback on institutional pathological diagnosis in a clinically relevant time frame. The UMBRELLA protocol addresses both Wilms tumour and non-Wilms tumours; in this Position Paper, we focus on the recommendations for Wilms tumours.

Box 1 Relevant aims of the SIOP–RTSG UMBRELLA 2016 protocol **Pathology aims**

To optimize pathological diagnostics by rapid central review in order to:

- Classify and stage tumours accurately in order to optimize treatment and avoid undertreatment or overtreatment.
- Monitor and give appropriate feedback on local pathological diagnosis and stage.
- Accurately assess the blastema in postchemotherapy specimens.
- Correlate blastema volume and biomarkers with event-free and overall survival.

**Biobanking aims**

- To store biological material from all participating centres.
- To show the feasibility of storing serial blood and urine samples and tumour and germline material at diagnosis and at specific time points during treatment for international collaborative studies.
- To establish national biobanks (if not yet present) and generate a study-wide virtual biobank for translational research.
- To test the feasibility of returning biomarker results to treatment centres within a clinically relevant time frame.

**Molecular biology aims**

- To assess genomic 1q gain as a prognostic marker in Wilms tumour.
- To analyse biomarkers with potential prognostic relevance (simultaneous loss of 1p and 16q, *MYCN* gain and 17p loss encompassing the *TP53* locus).
- To assess molecular characteristics of blastemal-type Wilms tumours.
- To explore whether aberrations in* WT1, CTNNB1, AMER1, TP53, MYCN, FBXW7, GPC3, MLLT1, DIS3L2, DICER1, DROSHA, DGCR8, SIX1,* or *SIX2* considerably affect event-free or overall survival.
- To explore circulating biomarkers (microRNA and DNA) in blood, and/or plasma, and/or urine and to support research on epigenetics, tumour cell culture, and/or organoids and xenografts.

SIOP–RTSG, Renal Tumour Study Group of the International Society of Paediatric Oncology.

## Guidelines for specimen handling

As in previous SIOP trials and studies^14^, the UMBRELLA protocol mandates preoperative chemotherapy on the basis of clinical and radiological diagnoses in most patients. Thus, pathological diagnosis is mainly based on pretreated nephrectomy specimens. Pretreatment biopsy is not routinely recommended. Percutaneous cutting needle biopsy (tru-cut biopsy) can be considered in young children with stage IV disease and in children >10 years of age, as the frequency of non-Wilms tumours (rhabdoid tumour of the kidney and renal cell carcinoma, respectively) is increased in these age groups^16^. Biopsies are particularly not advised in children <6 months of age and in fully cystic tumours; immediate surgery is recommended in these children. If performed, open wedge biopsy automatically upstages tumours to stage III, irrespective of other findings, as it is regarded as an (artificial) breach of the capsule (rupture). However, percutaneous cutting needle biopsies do not upstage tumour.

### Institutional pathologist’s role

The institutional pathologist samples and processes the fresh tumour according to the protocol. The surgical specimen should be worked up without delay to minimize degradation, especially of RNA. After visual inspection, weighing, measuring and photographing, the entire surface is inked. The specimen is bisected longitudinally, and the macroscopic appearance — specifically the percentage of necrotic areas — is recorded and photographed. Samples for biological studies are taken from viable and, if present, macroscopically distinct areas. The whole specimen is fixed in formalin for 24–48 hours and, then, at least one complete longitudinal slice of the whole specimen is sampled for histological evaluation to capture tumour heterogeneity^17^. Most importantly, a guide block of the selected samples from the tumours is made by the pathologist to assist tumour staging^17^ (Supplementary Figure 1).

## SIOP histological classification

Wilms tumour is a histologically heterogeneous embryonal tumour composed of blastemal, epithelial and stromal components^17^. In Europe, patients diagnosed with Wilms tumour are treated with chemotherapy for 4 or 6 weeks before surgery, depending on metastatic status^14^. Postoperatively, treatment stratification relies on overall and local stage, and also on histological classification into low-risk, intermediate-risk and high-risk Wilms tumours^18^. As nearly 40% of all relapses occur in children whose tumour was not histologically classified as high-risk Wilms tumour, a need to improve treatment stratification is evident^2,19,20^.

Histological assignment to the different risk groups is based on quantification of chemotherapy-induced changes, the percentages of the different viable Wilms tumour components (epithelial, blastemal and stromal) and the presence or absence of anaplasia^17,18^ (Table 1). Histological classification after preoperative chemotherapy defines three major subgroups of Wilms tumours: low-risk (completely necrotic Wilms tumour), high-risk (blastemal type and diffuse anaplasia) and intermediate-risk tumours (all other types)^17,18^. To correctly subclassify the Wilms tumour, the percentages of chemotherapy-induced changes and viable tumour components are assessed and taken into account as per the histological classification criteria^17,18^ (Table 1). The low-risk group includes Wilms tumours that become completely necrotic owing to preoperative treatment. Tumours in other risk groups are subclassified on the basis of viable tumour components. The intermediate-risk group includes the epithelial-type, stromal-type, mixed-type and regressive-type tumours and Wilms tumours with focal anaplasia (Supplementary Box 2). In the intermediate-risk group, in addition to histological subclassification, the tumour volume after preoperative chemotherapy (measured using imaging) is of importance for treatment stratification. If the tumour volume is >500 ml in stage II/III mixed-type, regressive-type, or focal anaplasia-type tumours, these tumours are considered to have an increased risk of poor outcome and are treated aggressively^3^. Some 5% of Wilms tumours are multifocal (both unilateral and bilateral tumours), and they are more difficult to assess. Currently, treatment of these tumours depends on other parameters, such as their histological type and response to preoperative chemotherapy, and the best way for a pathologist to manage them is to adopt two methods of analysis: one method is to assess all nodules together and calculate the percentages of nonviable and viable components as if they represented one nodule, and the other method is to assess them individually (also commenting on their size). The treatment decision should be made at multidisciplinary team meetings and in consultation with national or international lead experts.

**Table 1:** Histological criteria for Wilms tumour subtyping in SIOP pretreated patients

Tumour type^a^	Chemotherapy-induced change	Histological features (% of viable tumour)		
		Blastema	Epithelium	Stroma
Completely necrotic	100	0	0	0
Regressive	>66	0–100	0–100	0–100
Mixed	<66	0–65	0–65	0–65
Mixed	<66	11–65	0–89	0–89
Epithelial	<66	0–10	66–100	0–33
Stromal	<66	0–10	0–33	66–100
Blastemal	<66	66–100	0–33	0–33

SIOP, International Society of Paediatric Oncology. ^a^The presence of diffuse anaplasia in any of the tumour types supersedes the subtypes; focal anaplasia also needs to be specifically mentioned.

### Blastemal type and blastemal volume

The most important viable component to recognize in pretreated Wilms tumours is blastema, which is the most undifferentiated tumour component and is composed of primitive, undifferentiated cells that are arranged in no particular pattern. Blastemal-type Wilms tumour (in which >66% of the viable tumour consists of blastema in a tumour that is >33% viable) confers a worse prognosis and is, therefore, classified as a high-risk tumour according to the SIOP–2001 working classification^18^. These blastemal-type Wilms tumours were treated with more intensive postoperative treatment than other tumour types in the SIOP–2001 trial, resulting in improved outcomes^15^. This improvement highlights the need to identify high-risk, chemotherapy-resistant blastema to avoid unnecessary failure of first-line therapy, especially as doxorubicin is omitted in treatment of stage II and stage III intermediate-risk tumours^19^. A retrospective analysis of the SIOP–2001 trial data showed that a threshold of ~20 ml residual blastemal volume in localized Wilms tumour could be used to improve stratification^13,14^. An accurate recognition of blastema needs more objective criteria than are currently available to minimize interobserver variation and to enable institutional pathologists to make a correct diagnosis. The SIOP–RTSG agreed that the blastemal volume threshold at which the risk of relapse rises dramatically needs to be established before its implementation as a prognostic factor. Thus, one of the aims of the UMBRELLA protocol is to optimize the definition of high-risk, blastemal-type Wilms tumour.

In the UMBRELLA protocol, institutional pathologists will not be required to calculate the volume of blastema in tumours, as it is not currently a criterion for treatment stratification. However, the estimated percentages of the nonviable and viable components (especially blastema) will be recorded, and blastemal volume will then be calculated in the protocol database. This calculation will be based on both pathological and imaging measurements of the tumour.

### Anaplasia

Anaplasia has been recognized as a high-risk Wilms tumour feature for many years, and it confers a worse prognosis^14,18,21–24^. Anaplasia can occur in any component of Wilms tumours, and it can be focal or diffuse^21,22^. Importantly, in order to diagnose anaplasia, all three criteria need to be present: large atypical tripolar and/or multipolar mitotic figures; marked nuclear enlargement, with nuclear diameters at least three times those of adjacent cells; and hyperchromatic tumour cell nuclei^21,22^. Although the criteria for anaplasia are well established, in many circumstances diagnosis can be challenging, resulting in a considerable number of undiagnosed or overdiagnosed patients^25^. This misdiagnosis illustrates the value of rapid central pathology review for correct postoperative treatment stratification.

Focal anaplasia is defined as the presence of one or two foci showing the abovementioned nuclear criteria with sharp demarcation within the primary intrarenal tumour and without evidence of anaplasia or prominent nuclear atypia (nuclear unrest) in other areas^22^. By consensus of the SIOP–RTSG pathology panel, the size of the anaplastic focus does not exceed 15 mm (which was previously undefined). Wilms tumour with focal anaplasia is regarded as an intermediate-risk tumour in the UMBRELLA protocol, but the tumour should still be subclassified according to other components (for example, focal anaplasia in mixed-type Wilms tumour). If focal anaplasia is present in blastemal-type Wilms tumour, it is regarded as a high-risk tumour.

Diffuse anaplasia is defined as nonlocalized or multifocal anaplasia, focal anaplasia with marked nuclear unrest in the rest of the tumour, or anaplasia outside of the kidney (anaplastic tumour in intrarenal or extrarenal vessels, renal sinus, extracapsular sites or metastatic deposits). If anaplasia is present in a biopsy sample or other incomplete tumour sample, the diagnosis of diffuse anaplasia is warranted^22^.

### Histological classification of bilateral Wilms tumour

Synchronous bilateral Wilms tumours (stage V) occur in ~5–8% of patients, and these children are more likely to have an underlying genetic predisposition^26^. These patients are treated with preoperative chemotherapy for 6–12 weeks and nephron-sparing surgery (NSS) is considered by the surgical panel, taking into account tumour response to chemotherapy, to spare as much renal function as possible. Each tumour is subclassified and staged separately according to the histological classification and SIOP staging criteria. For multifocal tumours, the approach described above should be used.

### Histological classification of nephrogenic rests

Nephrogenic rests are foci of embryonal cells that persist after 36 weeks of gestation and are considered precursors of Wilms tumours. Nephrogenic rests are identified in 35–40% of patients with unilateral and >90% of patients with bilateral Wilms tumours and are often associated with different syndromes or anomalies such as overgrowth syndromes (hemihypertrophy and Beckwith–Wiedemann syndrome), Denys–Drash syndrome and Wilms tumour, aniridia, genitourinary abnormalities and mental retardation (WAGR) syndrome^27–30^. The two main types of nephrogenic rests are perilobar and intralobar rests. The term nephroblastomatosis is used to describe multiple, multifocal, or diffuse nephrogenic rests^27,28^. Panlobar nephroblastomatosis encompasses complete replacement of the renal lobe or cortex by nephrogenic rests and is recognized as a high-risk factor for developing Wilms tumour^31–33^. Prolonged chemotherapy is recommended in patients with nephroblastomatosis in the UMBRELLA protocol. Most patients are treated with NSS, as this therapy enables disease-free survival while sparing a maximum of healthy parenchyma in both kidneys^32,33^.

### Histological diagnosis of cystic partially differentiated nephroblastoma

Cystic partially differentiated nephroblastoma is a distinct variant of Wilms tumour that usually occurs in children <3 years of age and is composed entirely of cysts with septa without tumour nodules inside them^34^. Molecular studies have shown that cystic nephroma and cystic partially differentiated nephroblastoma are unrelated tumours^35^. Intermediate-risk or high-risk Wilms tumours can also present with numerous cysts, especially after preoperative chemotherapy, but they also contain solid, nodular areas^34^. The presence of numerous cysts in these tumours is of no prognostic significance^34^.

### Histological classification of primarily operated Wilms tumours

In rare instances, for example in children <6 months of age, immediate surgery is recommended in the UMBRELLA protocol, as opposed to preoperative chemotherapy. This course of action leads to a different histological classification, as chemotherapy-induced changes are not present. Of note, the presence of substantial amounts of viable blastema is not of prognostic significance in primarily operated Wilms tumours^14^. Histological classification after immediate nephrectomy is based only on the presence or absence of diffuse anaplasia: if absent, the Wilms tumour is classified as intermediate risk and if present, the tumour is high risk^14,17^.

## Histological staging

At diagnosis, the tumour is staged as localized (stages I–III), metastatic (stage IV), or bilateral (stage V) disease in order to decide on duration of preoperative chemotherapy^14^. The histological staging and treatment stratification of Wilms tumours after preoperative chemotherapy has changed to some extent in the UMBRELLA protocol on basis of the results of the previous trials and studies and by consensus of the SIOP–RSTG pathology panel^3,15,19,23^. More detailed definitions have been introduced to aid in correct staging by local pathologists (Box 2).

Box 2 Staging criteria in the SIOP UMBRELLA protocol **Stage I**

- Tumour is limited to the kidney.
- Tumour is present in the perirenal fat but is surrounded by a fibrous (pseudo)capsule. The (pseudo)capsule might be infiltrated by viable tumour, which does not reach the outer surface.
- Tumour might show protruding (botryoid) growth into the renal pelvis or the ureter but does not infiltrate their walls.
- The vessels or the soft tissues of the renal sinus are not involved by tumour. Intrarenal vessel involvement might be present.

**Stage II**

- Viable tumour is present in the perirenal fat and is not covered by a (pseudo)capsule, but is completely resected (resection margins are clear).
- Viable tumour infiltrates the soft tissues of the renal sinus.
- Viable tumour infiltrates blood and/or lymphatic vessels of the renal sinus or of the perirenal tissue, but it is completely resected.
- Viable tumour infiltrates the wall of the renal pelvis or of the ureter.
- Viable tumour infiltrates the vena cava or adjacent organs (except the adrenal gland) but is completely resected.

**Stage III**

- Viable tumour is present at a resection margin. Nonviable tumour or chemotherapy-induced changes present at a resection margin are not regarded as stage III.
- Abdominal lymph node involvement is present by either viable or nonviable tumour.
- Preoperative or intraoperative tumour rupture, if confirmed by microscopic examination (viable tumour at the surface of the specimen at the area of the rupture).
- Viable or nonviable tumour thrombus is present at resection margins of ureter, renal vein, or vena cava inferior (always discuss resection margins with the surgeon).
- Viable or nonviable tumour thrombus, which is attached to the inferior vena cava wall, is removed piecemeal by a surgeon.
- Wedge or open tumour biopsy before preoperative chemotherapy or surgery.
- Tumour implants (viable or nonviable) are found anywhere in the abdomen.
- Tumour (viable or nonviable) has penetrated through the peritoneal surface.

**Stage IV**

- Haematogenous metastases (for example, lung, liver, bone and brain) or lymph node metastases outside the abdominopelvic region.

**Stage V**

- Bilateral renal tumours at diagnosis. Each side should be substaged according to the above criteria.

### Stage I tumours

Stage I tumours are confined within the (pseudo)capsule and, even if the tumours are outside of the kidney in the perirenal fat but surrounded by the (pseudo)capsule and completely removed, they are regarded as stage I. The vessels or soft tissues of the renal sinus are not involved. The presence of necrotic tumour or chemotherapy-induced changes in the renal sinus, renal veins and/or within the perirenal fat is not a reason for upstaging. Botryoid tumour growth into the renal pelvis is also not a reason for upstaging the tumour. Furthermore, infiltration of the soft tissues between the kidney and the adrenal gland or of the adrenal gland itself does not cause upstaging of the tumour if the external capsule of the adrenal gland is intact. However, the presence of viable tumour in the lymphatic or blood vessels in this area is regarded as stage II. Tumour adhesion to the liver capsule is not regarded as infiltration of an adjacent organ; the tumour is upstaged (to stage II, if completely resected, or to stage III, if incompletely resected) only if clear infiltration of the liver parenchyma is present. Tumour attachment to other retroperitoneal organs, such as the colon, should be assessed in the same manner. Fine needle aspiration or percutaneous cutting needle biopsy (tru-cut biopsy) does not upstage the tumour.

### Stage II tumours

Stage II tumours are defined by viable tumour infiltration of the renal sinus (soft tissues and any vessels), and/or perirenal fat and/or adjacent organs (except the adrenal gland) with no clearly identifiable (pseudo)capsule but with clear resection margins. The presence of a viable tumour thrombus in the renal vein or inferior vena cava is also stage II if completely resected in one piece.

### Stage III tumours

Stage III tumours are defined according to a number of criteria (Box 2). Several of these criteria have undergone important changes in comparison with the SIOP–2001 trial criteria^18^. Now, in the UMBRELLA protocol, the presence of nonviable tumour or chemotherapy-induced changes only at a resection margin is not regarded as stage III. The finding of viable or nonviable tumour thrombus bulging out at the resection margin of the renal vein or inferior vena cava needs to be discussed with the surgeon. If the surgeon confirms they resected the vein away from the thrombus, then protruding thrombus at the vascular resection margin does not upstage the tumour to stage III. If the thrombus is removed with force, owing to attachment to the vascular wall, or by a piecemeal resection, stage III must be considered at a multidisciplinary meeting. The UMBRELLA protocol now explicitly states that the presence of prechemotherapy tumour rupture at diagnosis on imaging studies is only considered as pathological stage III if viable tumour is seen microscopically at the rupture site of the nephrectomy specimen. If not, the tumour is staged on the basis of the other pathological criteria, but the final treatment stage must be decided after discussion at a multidisciplinary team and/or tumour board meeting.

The presence of chemotherapy-induced changes (even without viable tumour) in a lymph node is regarded as proof of tumour metastasis and, therefore, the tumour is assigned stage III. Mature tubules can be found in lymph nodes, often associated with Tamm–Horsfall protein deposits, but this finding should not be considered as lymph node metastasis^36^. Accumulation of foamy macrophages within the subcapsular or interfollicular sinus should not be regarded as a metastasis. Nonviable metastasis is regarded as replacement of normal lymph node architecture with foamy macrophages and/or chemotherapy-induced changes.

The renal parenchyma should also be examined for the presence of nephrogenic rests and for indications of an underlying predisposing syndrome^37^. For example, patients with Denys–Drash syndrome often demonstrate diffuse mesangial glomerulosclerosis, and Beckwith–Wiedemann syndrome is associated with nephrolithiasis, medullary dysplasia and other renal abnormalities^38^.

### Staging in nephron-sparing surgery

Whenever NSS is pursued, assessing the resection margins very carefully is extremely important. Often, tumour nodules are resected with a small rim of renal parenchyma, especially in patients who have multiple nodules within one kidney or bilateral tumours. Procedures are encoded as NSS (A), which is partial nephrectomy (resection of tumour with a rim of normal renal parenchyma), and NSS (B), which is enucleation (resection of tumour without a rim of normal renal parenchyma)^39^. Histopathological examination in NSS includes evaluation of the complete circumference of the lesion. Small lesions are embedded completely. The minimal distance of the Wilms tumour to the resection margin will be measured. A safe rim has been defined as a margin of at least 1 mm, as is generally accepted in tumour pathology. The finding of nephrogenic rests at the resection margin is not regarded as a positive resection margin and does not upstage the Wilms tumour.

Histopathological assessment clearly states one of the following findings — safe rim of renal parenchyma on resection margin (except nephrogenic rests), intact (pseudo)capsule along the resection margin, or tumour breach and/or rupture.

## Rapid central pathology review

**Fig. 1:**
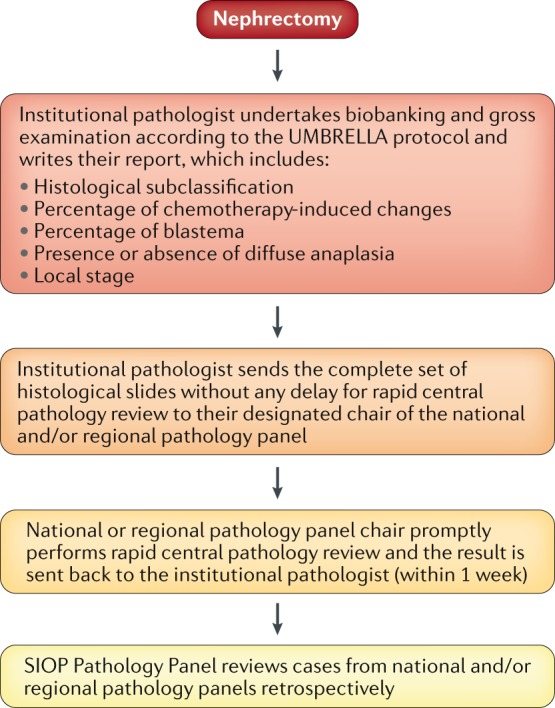
Flow diagram of rapid central pathology review. After nephrectomy, the institutional pathologist biobanks the specimen and examines it according to the UMBRELLA protocol, producing a report. The institutional pathologist then sends the complete set of slides for rapid central pathology review to their regional or national panel. The chair of the regional or national pathology panel promptly undertakes central pathology review and sends back their results to the institutional pathologist within 1 week. The International Society of Paediatric Oncology (SIOP) pathology panel reviews cases from the regional and national pathology panels restrospectively.

As in the previous SIOP trials and studies, all renal tumours and their assessment by the institutional pathologists will be reviewed by their national and/or regional pathology panel (Fig. 1). The results of previous Wilms tumour trials and studies have shown considerable discrepancies in diagnosis and staging of renal tumours between the institutional pathologists and central pathology review^25^. In the SIOP–2001 trial, rapid central pathology review was introduced in some parts of the trial, and it proved to be feasible and extremely valuable, avoiding undertreatment and overtreatment caused by misdiagnosis and/or mis-staging by institutional pathologists^25^. The SIOP–2001 study also resulted in an increase in submissions for central pathology review from 76% (in the UKW3 trial) to 100% in the UK part of the SIOP–2001 trial^25^.

Discrepancies in diagnosis and staging between institutional pathologists and central pathology review are likely to remain, but introduction of rapid central pathology review in the UMBRELLA protocol in more countries than it was available to in SIOP–2001 will enable clinicians to modify treatment in a timely manner, if required. Rapid central pathology review should be undertaken before postoperative treatment has started (which, according to the UMBRELLA treatment protocol, has to start 21 days after the last dose of preoperative chemotherapy). All participating centres will be required to send their samples to the dedicated review pathologist for central pathology review. As >50 countries will be participating in the UMBRELLA study, central pathology review will be provided by national and/or regional pathology panels (Fig. 1; Supplementary Table 1).

Additionally, all cases are peer reviewed by the international SIOP–RTSG pathology panel. This review is considered a quality control, as the individual pathologists do not receive a report from this panel.

## Biobanking and biomarker analysis

**Fig. 2:**
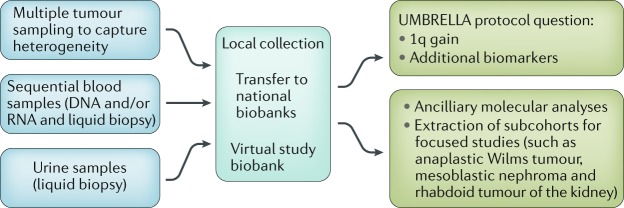
Biobanking and biomarkers in the UMBRELLA protocol. Tissue, blood and urine samples are collected locally and included in national biobanks that are virtually connected. These materials are primarily used to answer UMBRELLA protocol questions. Any surplus materials will be made available for ancillary and focused studies.

Two of the primary aims of the UMBRELLA protocol are biobanking and biomarker analysis (Box 1; Fig. 2). The biobanking aim is to demonstrate the feasibility of storing serial blood and urine samples, as well as tumour and germline material, at diagnosis and at specific time points during treatment for international collaborative studies. The molecular biology aim is to validate the prognostic value of 1q gain and other copy number alterations (1p/16q, *MYCN* and *TP53*) that were identified in the previous SIOP–2001 trial and to demonstrate feasibility of testing in a clinically relevant time frame^5–7,11,12,40^.

Achieving comprehensive collection of biomaterials in a uniform manner across the broad panel of participating countries will be challenging, but success in this goal will be key for future large-scale molecular profiling and implementation of biomarker testing. To achieve this aim, a standard for biobanking and for the submission of samples to the reference biological laboratories has been developed. Virtually linked biobanks will be created at a national level. These biobanks will be instrumental to reach the aims of the study, including evaluation of novel genomic driver candidates, informative microRNA (miRNA) patterns, epigenome analyses and panel sequencing that will be performed in national laboratories. Individual approaches will also cover in vitro and in vivo models and other non-Wilms renal tumours.

### Sample collection and storage

Sample collection requirements for the UMBRELLA protocol are divided into an essential minimal set and optional extensions to answer specific research questions. For baseline diagnostics, EDTA blood samples and tumour tissue together with healthy kidney tissue (if available) will be collected from primary tumours and relapses and stored at –80 °C. Wherever possible, additional materials including blood samples for miRNA analysis and liquid biopsy testing of circulating tumour DNA, as well as urine for proteomic analyses, should be collected at multiple time points. This collection will facilitate parallel and future translational research, which are detailed in the aims of the UMBRELLA protocol.

Blood samples will be asked for at diagnosis, after 2 weeks of chemotherapy and before surgery. In addition, collection of samples at follow-up time points of 1 week after surgery and at the end of treatment will be requested. Separation of plasma and the cellular fraction by centrifugation within <2 hours after venepuncture is preferred to enable liquid biopsy analysis of circulating tumour DNA, as this process might provide an attractive option for upfront diagnostics or follow-up monitoring. For miRNA studies, blood collected in PAXgene tubes, serum samples and urine samples are needed.

To guarantee correct histological staging, tumour sampling is performed by the institutional pathologist. Given the phenotypic and genetic heterogeneity of Wilms tumours, extensive sampling is needed^41^. Samples of ~1 cm^3^ each need to be selected from three or more spatially distinct sites within the tumour, as well as from macroscopically different lesions (if present) to capture potential heterogeneity. To make sure that representative and viable tissue has been selected in all samples, frozen sections or touch imprints can be prepared for evaluation of cellular content, viability and proportion of tumour cells. Besides tumour tissue, collecting matched normal tissue for comparative analyses from all patients is advised.

Snap freezing of tissue in liquid nitrogen must be done as soon as possible after excision to ensure minimal sample degradation. Duration from resection until freezing of the samples will be recorded for quality control. Importantly, all surgically removed metastases or relapses must be collected using the same protocol to facilitate identification of drivers of therapy resistance and metastatic potential. Biobanking of frozen materials will be complemented by surplus formalin-fixed, paraffin-embedded (FFPE) blocks that are also a valuable resource for research, including the molecular analysis of microdissected nephrogenic rests, tumour subcompartments and anaplastic foci.

Clinical data and biological samples will be collected on a national basis in the paediatric oncology centres where the children are treated. To achieve inclusion of all registered patients, each country will set up a network for the provision of biological samples. Such a network for submission and central storage of frozen tumour samples from ~80% of children is already established in the UK, Germany, the Netherlands, France and Italy. Sample collection in this virtual biobank, including medical records, informed consent for biobanking by parents and (if applicable) children, will be documented in the web-based SIOP UMBRELLA database.

The current proposal aims to further increase inclusion of multiple samples, especially from heterogeneous Wilms tumours, and to enable additional countries to provide the required samples and data. Depending on each national coordinating centre, frozen blood and urine samples can be either directly sent or stored on site until tissue samples are available for batched collection and shipping to the national biobank. Participating countries that lack appropriate infrastructure are encouraged to collect samples and forward batches to larger, host biobanks that can provide the necessary storage capacity and expertise in subsequent laboratory work-up.

### Biomarker analysis

Children’s Oncology Group (COG) protocols in the USA already include molecular markers in clinical decision making^14^. The results from our previous SIOP studies are equally encouraging but not yet definitive enough to mandate a change in clinical approaches^12,14^. With the even larger community of participating countries in the UMBRELLA protocol than in the SIOP–2001 study, we expect to collect sufficient materials for studies to provide a definitive answer on whether or not we need to include certain biomarkers in the SIOP protocol as well. The two primary biomarkers under study — blastemal volume and 1q gain — must be considered in the context of any co-dependencies with existing risk factors (tumour stage and histology) and the treatment given to prove their additional value for clinical decision making. On the basis of currently available data and agreed statistical parameters (power of 80% and a significance threshold of α = 0.05), up to 380 (for blastema volume) or 850 (for 1q gain) samples will be needed to test an association with outcome. This analysis entails a prospective duration of 5 years to collect the samples. In the case of 1q gain, testing will be performed on frozen and FFPE material to increase numbers, as several of the participating countries will not be able to guarantee successful provision of high-quality frozen samples.

No final decision has yet been made on the best approach to test for 1q gain and other copy number alterations. Multiplex ligation-dependent probe amplification analysis proved to be cost-effective and reliable in SIOP–2001 (ref.^12^), but the number of targets is limited. Single nucleotide polymorphism arrays or focused next-generation sequencing (NGS) panels might provide improved resolution to identify critical subregions, including copy number neutral allele loss.

Several additional genomic copy number biomarkers beyond 1q gain have potential prognostic relevance in Wilms tumour (Box 1). Simultaneous allele loss of 1p and 16q is associated with poor outcome in patients included in COG analyses treated with immediate nephrectomy, although the rarity of this aberration limits its usefulness^42^. *MYCN* gain was found to be associated with adverse outcomes in a retrospective SIOP–RTSG study published in 2015 (ref.^40^). Loss of p53 function might be associated with poor outcome in anaplastic tumours, which was suggested to be associated with the volume of anaplastic regions and their incomplete removal^5–7^.

Comprehensive NGS analysis of two Wilms tumour cohorts has yielded a large number of potential driver genes mutated in Wilms tumour, including *AMER1, BCOR, CTNNB1, DGCR8, DICER1, DIS3L2, DROSHA, FBXW7, GPC3, MLLT1, MYCN, SIX1, SIX2, TP53* and *WT1* (refs^8–10^). Evaluating whether aberrations in any of these genes, alone or in combination, have a considerable effect on clinical course and survival will be interesting. With the exception of *TP53*, mutations in individual known Wilms tumour genes have not generally been associated with adverse outcome. However, combined *SIX1* or *SIX2* mutations and microRNA processing pathway mutations did confer a worse outcome in at least one of two studies^8,9^. Thus, a planned targeted NGS panel for Wilms tumour will facilitate these biomarker studies.

The secondary molecular aims include a much broader scope of studies, covering exploratory analyses of potential novel biomarkers, including circulating nucleic acids detectable in blood and urine, for diagnosis and prognosis. Liquid biopsies, especially, offer the potential to provide a global view on the frequently heterogeneous tumours, reducing the risk of overlooking relevant tumour subclones.

On the national level, multiple efforts are underway to explore the role of miRNAs in blood and tumour tissue as biomarkers. A range of initiatives to molecularly characterize all subtypes of Wilms tumour and non-Wilms tumour and their associated nephrogenic rests using whole-genome, epigenomic and proteomic approaches have been planned. This characterization extends to in vitro and in vivo cell models, in which tumours are used to establish cell cultures, organoid cultures, or xenograft models that should support functional validation of newly discovered molecular aberrations and biomarkers and screening for novel therapeutic options.

Last but not least, the comprehensive collection of paediatric renal tumours will also aid in the assembly of sufficiently large cohorts to analyse rare entities such as mesoblastic nephroma, cystic partially differentiated nephroblastoma, clear cell sarcoma of the kidney, rhabdoid tumour of the kidney, or renal cell carcinoma, for which the molecular basis is often not known. This analysis might also uncover genetic alterations that can be assayed by liquid biopsies, generating future options for upfront noninvasive discrimination of these tumours and paving the way for improved therapeutic approaches that might lead to either omission of chemotherapy or a change to more appropriate drug regimens.

## Conclusions

Successful implementation of the UMBRELLA protocol will have the potential to shape future therapeutic approaches and to improve outcomes through several measures. The UMBRELLA protocol provides guidelines for clinical practice and will hopefully stimulate further international collaboration with harmonization of treatment protocols and research. The UMBRELLA study will provide increased patient cohorts, and the rapid central pathology review, the standardization of specimen handling and the improved collection of biological samples by pathologists will provide large numbers of specimens with increased homogeneity compared with other studies collected prospectively. These improved procedures will be of utmost importance to validate biomarkers such as 1q gain, to find driver gene mutations, to evaluate the prognostic significance of blastemal volume, and to unravel tumour heterogeneity with the ultimate goal to improve treatment stratification and to delineate novel therapeutic targets.

## Supplementary information

Supplementary Information
