# Supplementary material for: The UMBRELLA SIOP–RTSG 2016 Wilms tumour pathology and molecular biology protocol
Source: Nat Rev Urol. 2018 Oct 11;15(11):693–701. doi: 10.1038/s41585-018-0100-3 (PMC7136175; doi:10.1038/s41585-018-0100-3)
Supplement: Supplementary file 1 — Supplementary Information [file 41585_2018_100_MOESM1_ESM.pdf]

nature REVIEWS UROLOGY

The UMBRELLA SIOP–RTSG 2016 Wilms tumour pathology and molecular biology protocol

Gordan M. Vujanić, Manfred Gessler, Ariadne H. A. G. Ooms, Paola Collini, Aurore Coulomb-l'Hermine, Ellen D'Hooghe, Ronald R. de Krijger, Daniela Perotti, Kathy Pritchard-Jones, Christian Vokuhl, Marry M. van den Heuvel-Eibrink and Norbert Graf, on behalf of the International Society of Paediatric Oncology–Renal Tumour Study Group (SIOP–RTSG)

https://doi.org/10.1038/s41585-018-0100-3
Corrected: Publisher Correction

SUPPLEMENTARY INFORMATION

Supplementary Figure 1. **Handling of specimen by pathologist**

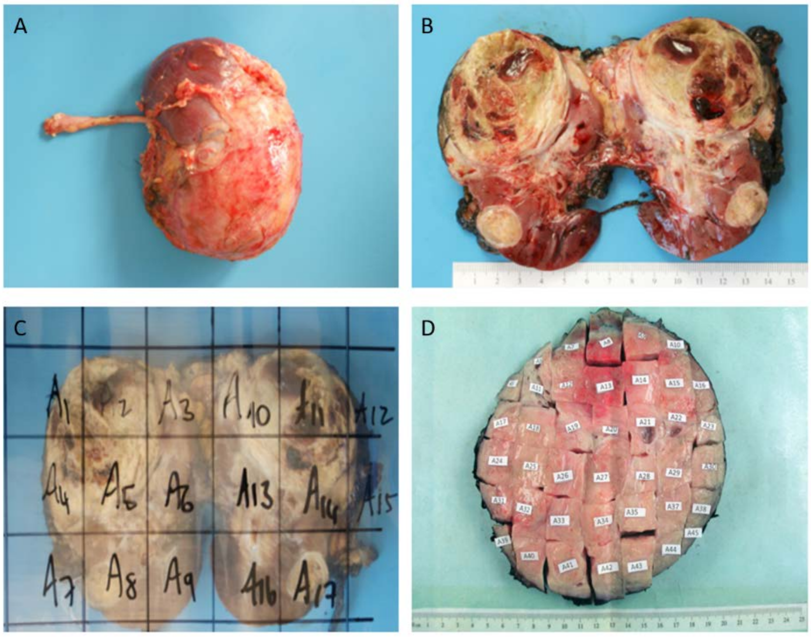

A: Fresh tumour on arrival at the pathology department

B: Bi-valved tumour after inking of the surface reveals a multifocal, partly cystic and haemorrhagic tumour that replaces most of the normal kidney parenchyma

C and D: examples of a guide block to indicate where each tumour block is sampled

Supplementary Box 1. HANDLING OF SPECIMENS BY THE PATHOLOGIST

Intact surgical specimens are delivered fresh to the pathology department without being incised by the surgeon. Preferably, the surgeon marks important areas that need attention, and accompanies the specimen to the pathology department to discuss the specimen with the pathologist directly. As soon as it arrives at the pathology department, processing starts in order to minimize degradation of DNA and RNA. The examination of the specimen includes:

1. **Weighing**, **measuring** and **photographing** the whole specimen (FIG. S1A). Look carefully for ruptures/fissures and ink them in a different colour then the rest of the specimen.
2. Search for and dissect the peri-renal and peri-hilar **lymph nodes**. Record the presence of (suspicion of) tumour and/or necrosis. Block these separately, recording their sites.
3. **Identify the renal vein, artery and ureter** and take transverse sections of each at/near the resection margin.
4. **Ink** the surface of the whole specimen and renal sinus with Indian ink. This is a critical step and always needs to be done, otherwise it might be impossible to stage the tumour accurately.
5. **Open** the specimen by a longitudinal incision (bi-valve) to demonstrate the tumour and its

relation to the kidney, capsule, and renal sinus (FIG. S1B).

6. **Photograph** the tumour, and record the macroscopic appearance.
7. **Measure** the tumour in all three dimensions, since this will be used for calculating volume.
8. **Assess the percentage of necrotic tumour.**
9. **Take samples required for biology studies** (described in the main text).
10. **Fix** the specimen in formalin (24-48 hours).
11. **Samples for histological examination** include at least one complete sampled longitudinal slice of tumour and kidney surface, accompanied by a **guide block** (FIG. S1C and S1D). If available, use mega-cassettes as it makes histological assessment easier.

In addition, sample the following:

a) Areas of the tumour that look different macroscopically.
b) Areas marked by surgeons (mainly for tumour margin assessment).
c) All lymph nodes, including the recording of viable tumour and necrosis.
d) Renal pelvis, pelvic fat, ureter, and sinus vessels; especially the renal vein is inspected for evidence of tumour thrombus.
e) Each nodule separated from the main mass (in multifocal tumours).
f) Tumour-kidney interface and tumour-kidney capsule.
g) Areas of the capsule or peri-renal fat with suspicion of tumour infiltration.
h) Adhesions of tumour to surrounding tissues.
i) At least 2 blocks of normal kidney parenchyma and blocks from abnormal looking areas in the remaining renal tissue.

It is strongly recommended to state all relevant histological findings with its corresponding block/slide number (for example, 'renal sinus invasion in block A7') as it makes central pathology review easier. It is recommended to make two sets of all sampled blocks at the same time, and send the second set for rapid central pathology review, even if only with a provisional report (final report can be emailed once it is ready).

Supplementary Box 2. HISTOLOGICAL CLASSIFICATION

Beckwith and Palmer's criteria for histological sub-typing of Wilms tumours state that one component has to comprise at least 2/3 (66%) of a tumour mass for the tumour to be sub-classified accordingly[1]. Pre-operative chemotherapy results in so-called 'chemotherapy-induced changes' in many Wilms tumours. Therefore, the criteria applicable to sub-classification of primarily operated tumours are modified to take these changes into account and distinguish three different prognostic sub-groups: low, intermediate, and high risk[2,3].

The low risk tumours are cystic partially differentiated nephroblastoma (treated by surgery only) and completely necrotic Wilms tumour. The five histological types of Wilms tumours included in the intermediate risk group are regressive type, epithelial type, stromal type, mixed type and focal anaplasia. High risk Wilms tumours are blastemal type and diffuse anaplasia type. All Wilms tumour types are shortly described in the following section. In order to sub-classify and stage Wilms tumour tumour, it is essential to sample tumour according to the UMBRELLA Study protocol, and examine an adequate number of slides. If only a few slides are available, the pathologist should only make the primary diagnosis but should not try to sub-classify tumour.

Completely necrotic Wilms tumour

The diagnosis of a completely necrotic Wilms tumour is made when the tumour shows only regressive and/or necrotic changes caused by chemotherapy, in the absence of any viable tumour tissue, especially nests of blastema. The presence of scattered mature tubules, stroma and very small groups of blastemal cells is allowed, as they may represent remnants of nephrogenic rests and is not regarded as viable tumour. The typical histological appearance of pre-treated Wilms tumours is a mixture of necrosis, fibrosis, fibromyxomatous stroma containing lipid- and/or hemosiderin-laden macrophages. Necrosis is most often of coagulative-type. To identify 'ghost' structures a reticulin stain may be useful. Although complete tumour necrosis makes histological diagnosis of any tumour impossible, in many cases 'ghost' tumour structures (mainly blastema, occasionally epithelial elements) can be recognised. In addition, the presence of nephrogenic rests is a very reliable clue that the tumour was a Wilms tumour. Finally, if treated with pre-operative chemotherapy for Wilms tumour, other renal tumours (such as clear cell sarcoma of the kidney, rhabdoid tumour of the kidney, renal cell carcinoma) show only minimal to moderate chemotherapy-induced changes[4].

Epithelial type Wilms tumour

The criteria for diagnosing epithelial type Wilms tumour are that the viable part of the tumour must comprise more than 1/3 of the tumour, and of the viable tumour, at least 2/3 consists of epithelial structures (TABLE 2). The rest of the tumour can be stromal and blastemal, but only up to 10% of blastemal component is allowed to be present (if >10% of blastema is present, the Wilms tumours are sub-classified as mixed type). Epithelial elements include tubules, rosettes, papillary structures, and glomerular structures. Epithelial Wilms tumours usually occur in younger children (median age 15 months), and about 80% of cases are in stage I[5].

Stromal type Wilms tumour

Stromal type Wilms tumour is diagnosed in tumours which are at least 1/3 viable, and the viable part consists of more than 2/3 of stromal elements. The rest of tumour may be epithelial and up to 10% blastemal (if >10% of blastema is present, such tumours are sub-classified as mixed type). The appearance of the stromal elements can be undifferentiated stromal cells, myxoid, fibroblastic, smooth muscle, skeletal muscle, adipose cells, cartilage and osteoid formation.

Stromal type Wilms tumour usually occurs in younger children[5]. However, stromal differentiation may be induced by pre-operative chemotherapy as a stromal type Wilms tumour is far more common in children who have received pre-operative chemotherapy[6]. Stromal type Wilms tumours usually show minimal to moderate chemotherapy-induced changes, since stromal tissue is usually resistant to chemotherapy[5,6].

Mixed type Wilms tumour

The histological criteria for making a diagnosis of mixed type nephroblastoma are: a) the viable part of the tumour comprises more than 1/3 of a tumour mass; b) the viable tumour consists of blastemal and/or epithelial and/or stromal elements, but none of them comprise more than 2/3 of the viable tumour; c) tumours which contain >10% of blastema, even if the predominant components are epithelial or stromal components.

Regressive type Wilms tumour

In regressive type Wilms tumour, the previously described chemotherapy-induced changes comprise more than 2/3 of the tumour mass, irrespectively of what the viable part of tumour is (except for diffuse anaplasia). The assessment of the percentage of necrosis/regression is done on both gross and histological examination. Therefore, blocks are taken not only from viable parts of the tumour mass, but also from the parts that show necrotic/regressive changes.

Wilms tumour with focal anaplasia

Wilms tumours which contain one or two foci of anaplasia according to the established criteria (see main text), are still sub-typed and should be reported as, for example, 'Wilms tumour, mixed type with focal anaplasia'. Generally, the size of an anaplastic focus should not exceed 15 mm. However, it is still important to determine the underling sub-type. If the underlying sub-type is blastemal, it is classified as a high risk tumour.

Blastemal type Wilms tumour

In blastemal type Wilms tumour, the viable part of the pre-treated tumour must comprise more than 1/3 of the tumour mass, and at least 2/3 of the viable tumour consists of blastema. The blastemal volume is discussed separately as a new item of the pathology protocol in the main text. The blastemal elements are composed of undifferentiated round or elongated cells, which are usually closely packed, and show no evidence of epithelial and/or stromal differentiation. There are several distinctive patterns (e.g. diffuse, serpentine) in which blastemal cells may occur, but they are of no prognostic or therapeutic significance.

Wilms tumour with diffuse anaplasia

Anaplasia occurs in about 5-8% of patients with Wilms tumour[1]. The finding of diffuse anaplasia over-rules the diagnosis of any other sub-type of Wilms tumour (for example, it may occur in regressive Wilms tumour), and always upgrades the Wilms tumour to the high risk category. The Wilms tumour with diffuse anaplasia as well as the distinction of focal versus diffuse anaplasia are described in the main article text.

REFERENCES - SUPPLEMENTARY INFORMATION

1. Beckwith, J. B. & Palmer, N. F. Histopathology and prognosis of Wilms tumors: results from the First National Wilms' Tumor Study. *Cancer* **41**, 1937-1948 (1978).
2. Delemarre, J. F., Sandstedt, B., Harms, D., Boccon-Gibod, L. & Vujanic, G. M. The new SIOP (Stockholm) working classification of renal tumours of childhood. *Med. Pediatr. Oncol.* **26**, 145-146 (1996).
3. Vujanic, G. M. *et al.* Revised International Society of Paediatric Oncology (SIOP) working classification of renal tumors of childhood. *Med. Pediatr. Oncol* **38**, 79-82 (2002).
4. Vujanic, G. M. & Sandstedt, B. The pathology of Wilms' tumour (nephroblastoma): the International Society of Paediatric Oncology approach. *J. Clin. Pathol.* **63**, 102-109,
5. Verschuur, A. C. *et al.* Stromal and epithelial predominant Wilms tumours have an excellent outcome: the SIOP 93 01 experience. *Pediatr. Blood &Cancer* **55**, 233-238, doi:10.1002/pbc.22496 (2010).
6. Weirich, A. *et al.* Clinical impact of histologic subtypes in localized non-anaplastic nephroblastoma treated according to the trial and study SIOP-9/GPOH. *Ann. Oncol.* **12**, 311-319 (2001).

Supplementary Table 1. **Chairs of the National / Regional Pathology Panels for the UMBRELLA 2016 Study**

Country / Region	Pathology Panel Chair
Egypt	Naglaa el Kinaii
France (SFCE)	Aurore Coulomb-L'Hermine
Germany (GPOH)	Christian Vokuhl
Italy (AIEOP)	Paola Collini
Japan	Yukichi Tanaka / Hajime Okito
Northern European countries	Ellen D'Hooghe
Poland	Jozef Kobos
South America	Isabela Werneck Cunha
Spain (SEHOP)	Enrique de Alava
The Netherlands (DCOG)	Ronald de Krijger
United Kingdom (CCLG)	Gordan Vujanic
SIOP (all other countries)	Gordan Vujanic
